# Extracellular vesicle-enriched miRNA profiles across pregnancy in the MADRES cohort

**DOI:** 10.1371/journal.pone.0251259

**Published:** 2021-05-12

**Authors:** Helen Bermudez Foley, Caitlin G. Howe, Sandrah P. Eckel, Thomas Chavez, Lili Gevorkian, Eileen Granada Reyes, Bethany Kapanke, Danilo Martinez, Shanyan Xue, Shakira F. Suglia, Theresa M. Bastain, Carmen Marsit, Carrie V. Breton

**Affiliations:** 1 Department of Preventive Medicine, Keck School of Medicine, University of Southern California, Los Angeles, California, United States of America; 2 Department of Epidemiology, Rollins School of Public Health, Emory University, Atlanta, Georgia, United States of America; 3 Gangarosa Department of Environmental Health, Rollins School of Public Health, Emory University, Atlanta, Georgia, United States of America; University of Mississippi Medical Center, UNITED STATES

## Abstract

MicroRNA (miRNA) circulating in plasma have been proposed as biomarkers for a variety of conditions and diseases, including complications during pregnancy. During pregnancy, about 15–25% of maternal plasma exosomes, a small size-class of EVs, are hypothesized to originate in the placenta, and may play a role in communication between the fetus and mother. However, few studies have addressed changes in miRNA over the course of pregnancy with repeated measures, nor focused on diverse populations. We describe changes in miRNA in early and late pregnancy from the MADRES cohort of primarily low-income Hispanic women based in Los Angeles, CA. miRNA derived from extracellular-vesicles (EVs) were isolated from maternal blood plasma samples collected in early and late pregnancy. In this study, we identified 64 of 130 detectable miRNA which significantly increased with gestational age at the time of collection (GA), and 26 which decreased with GA. Possible fetal sex-specific associations were observed for 30 of these 90 significant miRNA. Predicted gene targets for miRNA significantly associated with GA were identified using MirDIP and were found to be enriched for Gene Ontology categories that included energetic and metabolic processes but were underrepresented in immune-related categories. Circulating EV-associated miRNA during pregnancy are likely important for maternal-fetal communication, and may play roles in supporting and maintaining a healthy pregnancy, given the changing needs of the fetus.

## Introduction

microRNA (miRNA) are small non-coding RNA (~22nt) which modulate gene expression [1, 2] and may be dysregulated in multiple diseases and conditions, including cancer, diabetes, and Alzheimer’s disease, as well as in normal development [3–5]. They are also sensitive to the environment, with altered profiles reported for a variety of different biofluids in response to toxicant exposures, including metals, bisphenol-A, and formaldehyde [6–8].

There is growing interest in the use of miRNA found in maternal plasma as markers of maternal and fetal health. Some miRNA likely have important roles in healthy pregnancies and normal fetal development, but most studies have focused on miRNA associated with gestational pathologies, such as preeclampsia, preterm birth, and infant outcomes after fetal alcohol exposure [9–11]. Inflammation and body mass index (BMI) are also associated with miRNA profile changes during pregnancy, and these miRNA profiles may be useful biomarkers for certain pregnancy complications [12, 13]. The shifting profiles of miRNA in maternal circulation likely reflect changes in miRNA released by the placenta [14]. For example, oncogenic and anti-apoptotic miRNA targets have been reported to dominate the first trimester, while cell differentiation targets were found to be prevalent in the third trimester [15].

Although miRNA are abundant intracellularly, miRNA are also present extracellularly, circulating encapsulated in exosomes (~50-150nm) and other extracellular vesicles (EVs, up to 1 μm), along with proteins and other biological molecules [16, 17]. During pregnancy, about 15–25% of circulating EVs are estimated to originate in placental tissues [13]. These EVs increase in maternal circulation throughout the first trimester [18] and have been hypothesized to play important roles in maternal-fetal communication [19]. However, the mediating “cargo” in these EVs have not been sufficiently investigated. miRNA are likely candidates for some of the effects of EVs: for example, exosomal miRNA from the placenta are capable of modulating gene expression in maternal target cells [20], are hypothesized to contribute to some immunosuppressive functions during pregnancy [21], and are known to assist in transfer of gene-modulatory information (miRNA and mRNA) between cells [16]. If these processes are altered by changes in miRNA expression, critical parts of a healthy pregnancy could be compromised. Indeed, altered circulating miRNA profiles have been associated with several pregnancy complications, including preeclampsia, gestational diabetes, and preterm birth [22–24]. Evidence for the roles of exosomal miRNA during pregnancy, including evidence for miRNA effects in mouse models, has been reviewed by Yang et al., suggesting that miRNA is not simply a biomarker, but is also an active participant with many regulatory roles [25].

Circulating miRNA profiles are also likely dynamic over the course of a normal pregnancy, due to the changing stages of placental development and pregnancy progression [15]. However, a limited number of studies have used repeated measures to investigate circulating EV-encapsulated miRNA as a potential mediator of the role exosomes and EVs play in normal pregnancies [19]. Previous research has established a global increase of circulating exosomes miRNA during pregnancy [18], but did not describe miRNA-level changes over time. Few studies have characterized maternal circulating EV-associated miRNA longitudinally in pregnancy and have not established miRNA profiles in pregnancy for health disparities groups. Therefore, in the current study we characterized changes in EV-associated miRNA across pregnancy in the MADRES cohort, a primarily low-income Hispanic population of women in Los Angeles, California [26].

## Results

The majority of pregnant women included in the current study were in their mid-twenties, and about a third were primigravida (36%). (Table 1) Approximately 33% of participants were overweight and 35% were obese. Most participants’ preferred language was English (62%), and the majority of participants self-reported as Hispanic (75%). Among the self-identified Hispanic participants identifying country of birth, 55% were born abroad and 45% were born in the U.S. Maternal education was also an important characteristic of this population, with a large percentage completing no more than a high school education (58%).

**Table 1:** Demographics of the miRNA pregnancy cohort.

		Full Dataset		Paired-Sample Sensitivity Analysis	
		n	Mean (SD)	n	Mean (SD)
Maternal Age	Standardized at 12 weeks	337	28.5 (6.1) years	154	28.5 (5.9)
Pregnancy timing	Early pregnancy samples	192	13.6 (4.2) weeks	154	13.6 (4.1)
	Late pregnancy samples	299	31.7 (2.0) weeks	154	31.9 (1.8)
		n	Percent	n	Percent
Gestational Age at Birth	Preterm (<37 weeks)	35	10.4%	13	8.4%
	Term (37–40 weeks)	210	62.3%	100	64.9%
	Late term (40–42 weeks)	88	26.1%	39	25.3%
Fetal Sex	Female	161	48.2%	65	42.8%
	Male	173	51.8%	87	57.2%
Parity	Nulliparous	116	35.7%	50	32.5%
	Primiparous or higher	209	64.3%	104	67.5%
Language	English	191	62.4%	90	60.4%
	Spanish	115	37.6%	59	39.6%
Race/Ethnicity	US-Born White Hispanic	114	33.8%	51	33.1%
	Foreign-Born White Hispanic	139	41.2%	76	49.4%
	Other Hispanic	24	7.1%	4	2.6%
	Black Non-Hispanic	34	10.1%	15	9.7%
	Non-Hispanic Other	26	7.7%	8	5.2%
Education	Less than 12th grade (Did not finish high school)	99	29.4%	39	25.3%
	Completed 12th grade (Graduated high school)	97	28.8%	53	34.4%
	Some college or completed college	141	41.6%	62	40.3%
Pre-pregnancy BMI	Underweight or Normal Weight (< 25 kg/m^2^)	107	31.8%	49	31.8%
	Overweight (25 kg/m2–29.9 kg/m^2^)	111	33.2%	50	32.5%
	Class 1–3 Obese (> 30 kg/m^2^)	119	34.9%	55	35.7%

Notes: Samples may not add to 100% due to missingness in questionnaires. GA at birth may be unavailable for participants lost to follow-up at birth, however best estimated GA during pregnancy was available as described in the methods. Statistical tests (T-test: Maternal Age; Chi-square test: fetal sex, birth order, language, education, pre-pregnancy BMI; Fisher’s Exact test: Race) comparing the groups with 1 or 2 samples (full dataset) vs. 2 samples (sensitivity analysis) showed no significant difference for maternal age, fetal sex, birth order, language, race, education or BMI (p>0.05).

**Fig 1:**
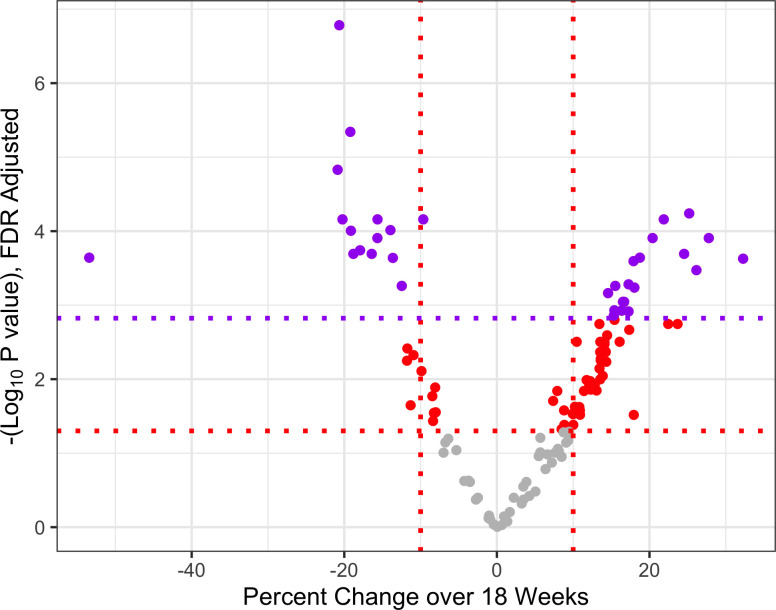
Volcano plot of miRNA associated with GA, scaled to 18 weeks of pregnancy. Red vertical dotted lines indicate -10% and 10% change over 18 weeks. Red and purple horizontal dotted lines indicate FDR-adjusted and Bonferroni-adjusted significance thresholds, respectively.

Covariate-adjusted linear regression models using all samples (n = 449 observations) identified 90 miRNA that were associated with gestational age (GA) after multiple testing correction (FDR P<0.05, Fig 1, S1 Table in S1 File). Samples with missing covariate data were excluded using the lme algorithm. GA was associated with an increase in miRNA counts during pregnancy for 64 of the miRNAs and with a decrease in miRNA counts for 26 of the miRNA, although individual participants may have had different trends (S5 Fig in S1 File).

The same 90 miRNA were associated with GA (FDR P<0.05) when restricting the analysis to participants who provided plasma samples at both timepoints (n = 296 observations), and all 90 changed in the same direction in both analyses (S1 Fig and S2 Table in S1 File). An additional 14 miRNA were also found to be associated with GA that were not identified in the primary analysis (S1 Fig and S2 Table in S1 File). A second sensitivity study was performed excluding pre-term births (<37 weeks, n = 399 observations). Eighty-six of the 90 miRNA identified in the main analysis remained significant, including the top miRNA, and just 4 miRNA lost significance (hsa-miR-1197, hsa-miR-151a-3p, hsa-miR-26a-5p, hsa-miR-369-3p). Eight miRNA were associated with GA in the sensitivity study, but not in the main analysis (S4 Table in S1 File).

Differences by fetal sex were also explored. When analyses were stratified by fetal sex, 48 miRNA were associated with GA in females, and 96 miRNA were associated with GA in males (FDR P<0.05) (S3 Table in S1 File). Of those, 33 miRNA were shared between pregnancies with male and female fetuses. However, none of the interaction terms for GA and fetal sex were statistically significant (FDR P>0.05).

**Fig 2:**
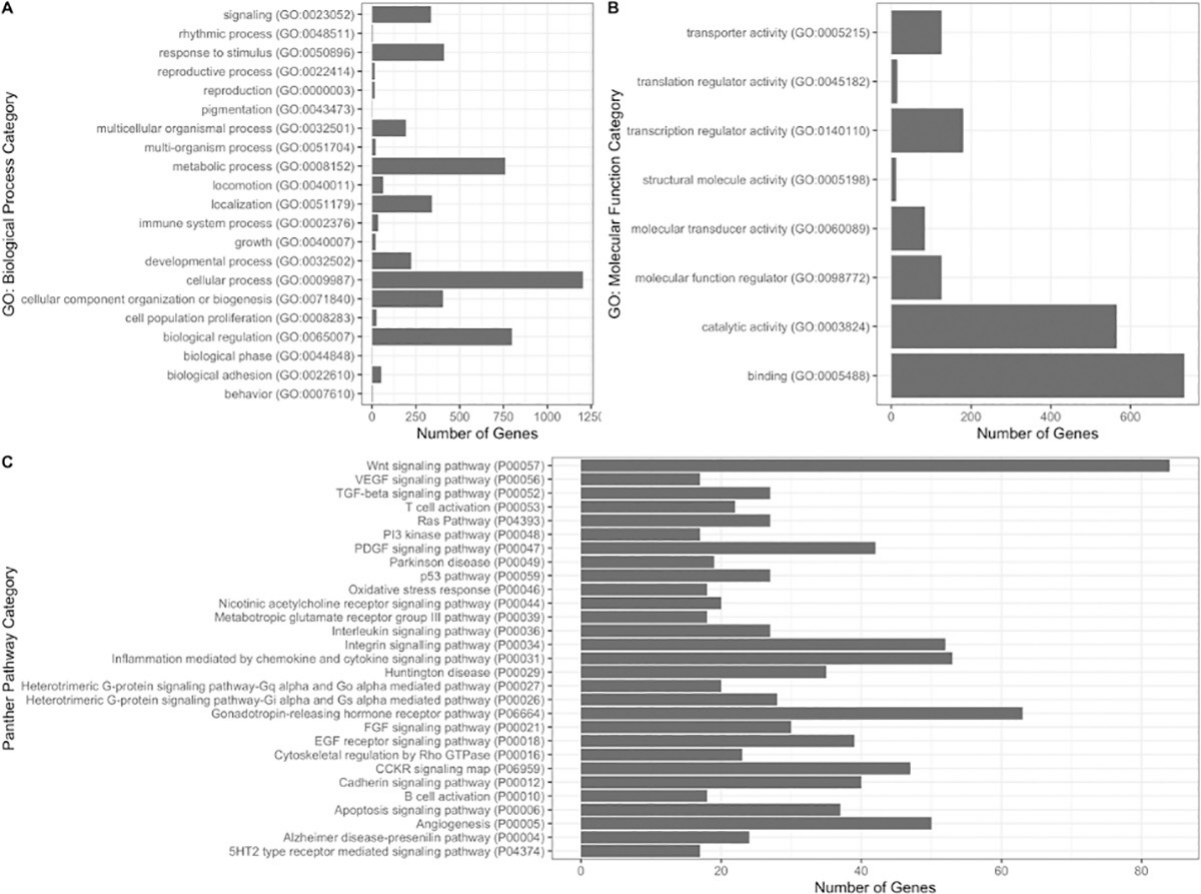
Distribution of genes in Gene Ontology categories. A. Number of genes in GO Biological Categories. B. Number of genes in GO Molecular Function. C. Number of Genes in PANTHER Pathways.

To investigate possible gene pathways related to the GA-associated miRNA, potential target genes were first identified using mirDIP using the 90 miRNA that were associated with GA in the primary analysis. Overrepresented and underrepresented PANTHER pathways and Gene Ontology categories (GOslim Biological Process and Molecular Function) are listed in Table 2. The top underrepresented categories in GOslim Biological Process were enriched in immune response categories. The PANTHER pathway analysis indicated enrichment of genes involved in signaling pathways, including Wnt, Ras, and EGF pathways. Large numbers of genes were associated with certain GO categories and pathways, especially those in metabolic processes, catalytic activity, binding and signaling pathways (Fig 2).

**Table 2:** Gene Ontology for predicted targets of miRNA significantly associated with GA. The first ten results for gene ontology (GOSlims Biological Process, GOSlims Molecular Function, PANTHER pathway classification) tests for significantly over- and under-represented groups (PANTHER overrepresentation test, FDR P<0.05) are presented in Table 2. No named PANTHER pathways were significantly underrepresented (FDR P>0.05).

**GOSlim Biological Process**	**Over**	**Fold Enrichment**	**Raw P-value**	**FDR adj. P-value**
regulation of primary metabolic process (GO:0080090)	+	1.71	1.49E-21	3.08E-18
regulation of nitrogen compound metabolic process (GO:0051171)	+	1.71	3.61E-21	3.72E-18
regulation of cellular metabolic process (GO:0031323)	+	1.69	7.84E-21	5.39E-18
regulation of macromolecule metabolic process (GO:0060255)	+	1.67	1.63E-20	6.73E-18
regulation of metabolic process (GO:0019222)	+	1.66	1.60E-20	8.24E-18
biological regulation (GO:0065007)	+	1.35	3.75E-18	1.29E-15
cellular macromolecule metabolic process (GO:0044260)	+	1.44	1.01E-17	2.61E-15
regulation of cellular process (GO:0050794)	+	1.39	9.24E-18	2.72E-15
regulation of biological process (GO:0050789)	+	1.37	4.30E-17	9.87E-15
regulation of nucleobase-containing compound metabolic process (GO:0019219)	+	1.72	9.00E-16	1.86E-13
**GOSlim Biological Process**	**Under**	**Fold Enrichment**	**Raw P-value**	**FDR adj. P-value**
immune response (GO:0006955)	-	0.35	5.82E-08	1.74E-06
immune effector process (GO:0002252)	-	0.08	1.26E-07	3.47E-06
defense response to bacterium (GO:0042742)	-	< 0.01	2.37E-07	5.88E-06
defense response to other organism (GO:0098542)	-	0.2	3.99E-07	9.04E-06
regulation of immune response (GO:0050776)	-	0.15	4.54E-07	1.01E-05
positive regulation of immune response (GO:0050778)	-	0.16	9.46E-07	1.95E-05
leukocyte mediated immunity (GO:0002443)	-	< 0.01	1.17E-06	2.30E-05
adaptive immune response based on somatic recombination of immune receptors built from immunoglobulin superfamily domains (GO:0002460)	-	< 0.01	1.16E-06	2.31E-05
activation of immune response (GO:0002253)	-	0.13	1.36E-06	2.56E-05
lymphocyte mediated immunity (GO:0002449)	-	< 0.01	1.79E-06	3.30E-05
**GOSlim Molecular Function**	**Over**	**Fold Enrichment**	**Raw P-value**	**FDR adj. P-value**
phosphotransferase activity; alcohol group as acceptor (GO:0016773)	+	2.04	1.22E-12	6.49E-10
protein kinase activity (GO:0004672)	+	2.12	4.68E-12	1.24E-09
catalytic activity; acting on a protein (GO:0140096)	+	1.56	8.28E-12	1.47E-09
protein serine/threonine kinase activity (GO:0004674)	+	2.21	2.27E-11	3.02E-09
kinase activity (GO:0016301)	+	1.92	3.31E-11	3.52E-09
transferase activity (GO:0016740)	+	1.54	4.88E-11	4.33E-09
protein binding (GO:0005515)	+	1.37	4.48E-10	3.41E-08
transferase activity; transferring phosphorus-containing groups (GO:0016772)	+	1.76	8.87E-10	4.72E-08
molecular function (GO:0003674)	+	1.16	8.34E-10	5.54E-08
transcription regulator activity (GO:0140110)	+	1.67	1.60E-09	7.74E-08
**GOSlim Molecular Function**	**Under**	**Fold Enrichment**	**Raw P-value**	**FDR adj. P-value**
structural constituent of ribosome (GO:0003735)	-	0.13	8.65E-05	0.00177
transmembrane signaling receptor activity (GO:0004888)	-	0.61	0.000304	0.00506
signaling receptor activity (GO:0038023)	-	0.68	0.00127	0.0161
structural molecule activity (GO:0005198)	-	0.42	0.00168	0.0203
G protein-coupled receptor activity (GO:0004930)	-	0.53	0.00596	0.0488
**PANTHER Pathways**	**Over**	**Fold Enrichment**	**Raw P-value**	**FDR adj. P-value**
Wnt signaling pathway (P00057)	+	2	1.02E-07	8.37E-06
Gonadotropin-releasing hormone receptor pathway (P06664)	+	2.05	2.38E-06	0.00013
Angiogenesis (P00005)	+	2.2	4.41E-06	0.000181
Integrin signaling pathway (P00034)	+	2.06	1.28E-05	0.000349
Apoptosis signaling pathway (P00006)	+	2.43	1.11E-05	0.000364
CCKR signaling map (P06959)	+	2.07	3.07E-05	0.00056
Ras Pathway (P04393)	+	2.76	2.91E-05	0.000597
PDGF signaling pathway (P00047)	+	2.19	2.83E-05	0.000663
EGF receptor signaling pathway (P00018)	+	2.17	7.23E-05	0.00119
Insulin/IGF pathway-mitogen activated protein kinase kinase/MAP kinase cascade (P00032)	+	3.66	0.000147	0.00219

P-values adjusted with FDR, p<0.05.

## Discussion

The data presented in this paper include a large number of participants with repeated measures during pregnancy, and focused on a predominantly lower-income population, many of whom have Hispanic heritage, and have historically been underrepresented in miRNA research. Thus, this study contributes a great deal to the diversity of currently available data for miRNA in pregnancy. A wide-range of pre-pregnancy BMIs, maternal education levels, and different racial groups were also represented in this study population. We identified 90 miRNA that were associated with GA, with the majority increasing over the course of pregnancy. Pathway analyses suggest that the gene targets of these miRNA are enriched in categories describing RNA and DNA regulation and are underrepresented in categories relating to immune response.

### miRNA detection

Of nearly 800 miRNA assayed, 130 miRNA were detectable above the sample-specific background in 50% of maternal plasma samples. This is the largest characterization of miRNA from EV-enriched samples during pregnancy. Interestingly, the majority of miRNA detected were also significantly associated with GA at the time of sample collection (90/130), indicating that many miRNA may play some role during pregnancy, although few of them have been previously assessed in studies focusing on pregnancy complications.

### miRNA associated with GA

Several studies have reported associations between miRNA, including EV-associated miRNA, and pregnancy related outcomes or conditions. However, much less is known about the role of EV-associated miRNA in normal development during pregnancy. Pregnancy is a complex and dynamic period. Maternal physiology must undergo substantial changes to meet the needs of the developing fetus, and it has been hypothesized that miRNA may contribute to coordinating these changes, in part by facilitating maternal-fetal communication during pregnancy [27]. miRNA profiles are therefore likely to differ between early and late pregnancy, and prior evidence suggests that oncogenic and anti-apoptotic miRNA targets may dominate the first trimester, while cell differentiation targets may be more prevalent in the third trimester [15].

In the current study, we identified a set of 90 EV-associated miRNA which were significantly associated with GA after adjustment for covariates, suggesting that these miRNA may change during pregnancy. In sensitivity analyses, we restricted participants with paired early and late pregnancy EV miRNA measures, and results were largely consistent with the main analysis.

While the majority of these miRNA (64) were positively associated with GA, which suggests that they increase over the course of pregnancy, we also identified a set of 26 miRNA that were negatively associated with GA. These associations may reflect some of the shifting responses and physiologic changes that occur during pregnancy. miRNA that decreased over the course of pregnancy may play a larger role at the beginning of pregnancy, while those that increase over the course of pregnancy may be important for supporting processes that occur in late pregnancy.

For example, GA was associated with lower levels of both miR-126-3p (raw P = 1.27E-09, FDR P = 1.65E-07) and miR-191-5p (raw P = 7.60E-06, FDR P = 9.87E-05). These two miRNA are known to regulate angiogenesis and may inhibit the development of new blood vessels or contribute to vascular remodeling critical to early placental growth [28–30]. Reduced maternal levels of miR-126-3p are hypothesized to contribute to vascular inflammation during pregnancy and have been reported at low levels in women with preeclampsia [31]. The miRNA analysis presented here did not exclude participants with pregnancy-induced hypertension or preeclampsia, however our results suggest that even in largely normal pregnancies, miRNA plays a role in vascular remodeling in early pregnancy and may also decrease in later pregnancy.

GA was also associated with lower miR-181a-5p counts (raw P = 7.01E-08, FDR P = 4.55E-06), and previous research suggests that this miRNA may be involved in the suppression of labor until later stages of pregnancy [32]. Decreasing levels of miR-181a, as observed in this study, are consistent with its role in supporting the beginning of labor. Low levels of miR-181a have also been associated with increased insulin resistance in non-pregnant obese subjects [33]. Reductions in this miRNA across gestation may therefore contribute to the increases in insulin resistance observed during pregnancy, in a manner similar to obesity [34]. Interestingly, miR-181a is also significantly decreased in newborns born to obese mothers [35], suggesting the mother’s metabolic status may influence the metabolic status of the newborn. The MADRES cohort has a high prevalence of pre-pregnancy obesity: 24% meet BMI criteria for overweight and 25% of mothers meet BMI criteria for obesity. Future research on metabolic health of expectant mothers and their children in the MADRES study is therefore critical.

Sixty-four of the 90 miRNA significantly associated with GA were positively associated with GA, and 34 were also found to be significant using Bonferroni correction. These included miR-1323 (raw P = 3.28E-06, FDR P = 6.93E-05), a placenta-specific miRNA, and miR210-3p (raw P = 1.08E-05, FDR P = 1.24E-04), which may play important roles in maintaining a healthy fetal weight and supporting placental function during pregnancy [36, 37]. Maternal plasma levels of miR-1323 were found to be lower in cases of fetal growth restriction, however the target pathways are unknown [36], and miR-210-3p expression in plasma was found to be lower for women with preeclampsia, acting through target genes that regulate trophoblast invasion and migration [37].

Interestingly, few of the miRNA from the chromosome 19 miRNA cluster (C19MC) and the chromosome 14 miRNA cluster (C14MC) that are particularly expressed during pregnancy were identified as significantly associated with GA [38, 39]. Several of these have been associated with pre-eclampsia (miR-518c, miR 525-5p, miR-526a, miR-299, miR-495 [40–42]), fetal growth restriction (miR 525-5p, [43]) and gestational diabetes (miR-518d, miR-1323 [44, 45]). Most of these have strong expression in the placenta, but have also been identified in circulating maternal plasma and in exosomes[14, 43, 45–48].

Other miRNA identified in this study have also been previously examined in maternal plasma and placenta. miR15a-5p is upregulated in exosomes isolated from maternal plasma in women with preeclampsia [49, 50] and miR 199a-5p in placenta is downregulated in cases of severe fetal growth restriction [51]. However, miRNA have multifaceted roles. Plasma and serum levels of miR127-3p increased in women with recurrent miscarriage [52], but increased levels during second trimester were associated with lower odds of a small for GA infant [53]. Although these roles appear contradictory, they may instead indicate that miRNA actions differ by situation, underscoring that studies addressing dynamics of miRNA profiles are critical for understanding the complex roles played by miRNA.

### Sex-specific associations for miRNA and GA

Previous research has largely overlooked differences in miRNA by fetal sex during pregnancy [11, 14, 15, 35–37, 54, 55]. In sex-stratified analyses, we found some evidence for possible differences by fetal sex for a subset of the miRNA. For example, 15 miRNA were associated with GA in males but not females, and another 15 miRNA were associated with GA in females but not males, while 33 were found to be similarly associated with GA in both males and females. However, cross-product interaction terms for GA and fetal sex were not statistically significant. Further research on interactions between fetal sex and miRNA during pregnancy is therefore critical for a more complete understanding of potential sex differences in miRNA changes during normal pregnancies.

### Gene target analysis

A single miRNA can affect the transcription of multiple gene targets. In fact, more than 30% of human genes are estimated to be regulated by miRNA [56]. Thus, even small changes in miRNA may have large impacts on genes involved in a variety of different pathways. EV-associated miRNA may exert particularly widespread effects given their ability to reach distal target tissues [27]. Results from our pathway analysis suggest that EV-associated miRNA in pregnancy may impact genes involved in major signaling systems, such as the Wnt and EGF signaling pathways, which are critical for development [57, 58]. Aberrant signaling in these pathways has been associated with pregnancy complications, such as gestational diabetes, and are targets of disrupted miRNA in pregnancy [59, 60]. Other significant pathways identified in our analysis included the cadherin and PGDF signaling pathways, which are associated with cell differentiation and regulation of cellular growth, consistent with the major changes in maternal and fetal growth that occur during pregnancy.

Gene Ontology results were similar. The gene targets of miRNA associated with GA were found to be enriched in categories describing RNA and DNA regulation, suggesting that miRNA in pregnancy could affect transcription factors in feedback loops that may amplify downstream effects, given small changes in miRNA number [61]. Transferase and kinase activity may also indicate changing energy and ATP usage. Interestingly, the gene targets of these miRNA are underrepresented in categories relating to immune responses, meaning that fewer genes than expected for these miRNA are involved in immune responses as they change over pregnancy (GA). The importance of miRNA in supporting healthy placentation in early pregnancy and immunotolerance during pregnancy is well-supported [62–64]. In particular, miR-223, miR-155 and miR146a are known to support regulatory T-cells that reduce inflammation and reduce cell-mediated immunity in early pregnancy [65]. In this data, miR-223 and miR-146a were negatively associated with GA, suggesting that some miRNA involved in immune tolerance may change with GA although GA-associated miRNA are generally not involved with the maternal immune response.

### Strengths and limitations

In this study we examined how EV-associated miRNA profiles in maternal plasma change during pregnancy in a cohort of predominantly low-income Hispanic women, contributing to the literature on miRNA in pregnancy. The data presented here reflect a large array of miRNA screened (nearly 800 unique miRNA), more than is feasible with standard qPCR techniques, and characterizes miRNA associated with GA.

Although the samples in this study were collected during both early and late pregnancy, reflecting changes in miRNA across the course of pregnancy, the data reported here did not have the temporal resolution to describe the trajectory of miRNA expression in finer detail. Identification of miRNA associated with fetal sex may have been limited by smaller sample sizes but warrants further investigation. While this paper does not establish a baseline for normal miRNA expression throughout pregnancy, the data suggest that caution should be used in interpreting miRNA at different timepoints of pregnancy. While the full cohort included 35 preterm births, a sensitivity analysis in which they were removed found little difference in miRNA associated with GA in this population.

The EV miRNA extraction method we used included a size- selection step which enriched for exosomes, including placenta-derived exosomes, so these measures may also include miRNA from other circulating EV subtypes. The methods used did not specifically focus on placenta-derived EVs and miRNA, but it is likely that miRNA assessed in this study include both placenta-derived miRNA (estimated at 15–25% of all maternal circulating EVs [13]) as well as encapsulated maternal miRNA. Additionally, because this study lacks pre-pregnancy miRNA data on these participants, it is possible that some miRNA are not expressed exclusively during pregnancy, but generally present in women and may be affected by pregnancy status and GA. Combining EV classes and size fractions may have reduced the signal of maternal-fetal crosstalk or sex-specific miRNA expression that could be limited to a particular size class of EVs. Further investigation into the functional roles miRNA selected to different EV size classes may yield more details about maternal-fetal crosstalk and the mechanisms of miRNA action during pregnancy. Additional data on promising miRNA for use as biomarkers could be studied with more timepoints by RT-qPCR, establishing in a better baseline for future clinical use.

Finally, pathway analysis is also a challenging area for studies of miRNA in pregnancy, often relying on cancer-related data that share some mechanisms, but are somewhat unclear in interpretation [66, 67]. In order to address the complexity of miRNA predictive analysis, we limited analysis to GOslims categories, to reduce the number of associations with cancer-related pathways, and still found that the predicted genes reflected many characteristics that match with pathways hypothesized to change during pregnancy.

## Conclusion

EV-associated miRNA that change in expression during pregnancy may be involved in important cell signaling and transcriptional regulation pathways that are critical to supporting a healthy pregnancy. A greater understanding of the biological role of miRNAs during pregnancy may help to develop biomarkers for pregnancy and birth complications through the use of blood or other non-invasive biofluids that would help to improve both maternal and children’s health trajectories [12, 50, 68, 69]. Previous studies on miRNA during pregnancy have been limited by small sample sizes, the quantification of a few select miRNA, and the omission of data of racial or ethnic background, and other key demographics of the participants, such as pre-pregnancy BMI.

Data from diverse populations are critical for understanding the normal roles of miRNA in pregnancy and how differences in underlying health conditions may affect pregnancy. Certain populations are at increased risk for metabolic disorders, such as obesity and diabetes, due to socio-cultural and economic backgrounds. Metabolic conditions are known to alter miRNA profiles [33, 70, 71], and thus associations between miRNA and underlying conditions must be continue to be studied in these at-risk groups. EV-enriched miRNA profiles during pregnancy in diverse study populations will help to improve the development of early and minimally invasive biomarkers that can be used to improve maternal and child health outcomes.

## Materials and methods

### Participants

**Fig 3:**
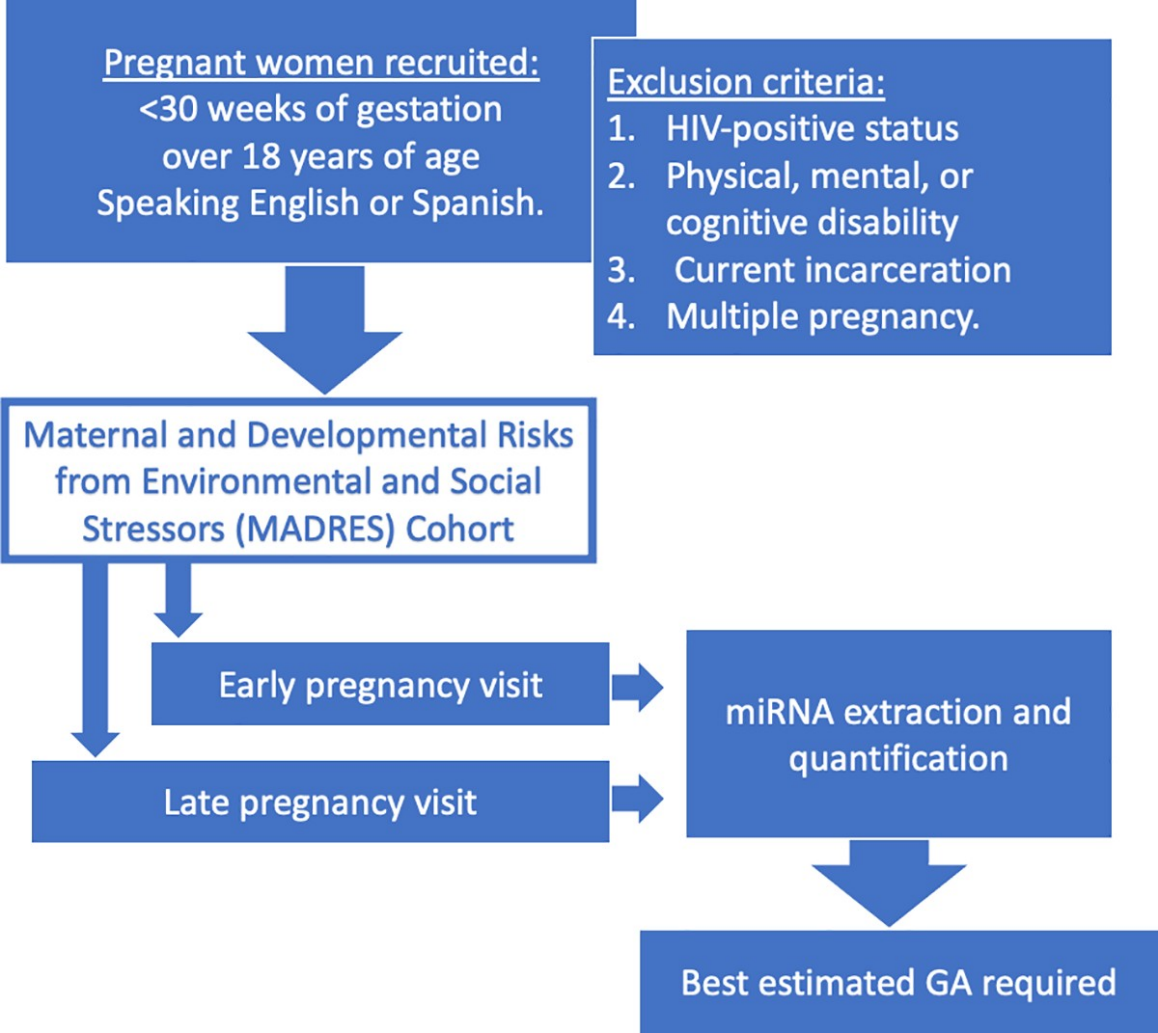
Recruitment and study flow chart.

The Maternal and Developmental Risks from Environmental and Social Stressors (MADRES) study is an ongoing cohort of more than 800 pregnant women and their children receiving prenatal care in Los Angeles, CA (USA). The full study design and cohort description is available in an earlier publication [26]. Pregnant women were recruited to the study at less than 30 weeks of gestation, were over 18 years of age, and spoke English or Spanish. Participants with the following conditions were excluded: 1. HIV-positive status, 2. physical, mental, or cognitive disability preventing participation, 3. current incarceration, and 4. multiple pregnancy. Study procedures were approved by the University of Southern California Institutional Review Board (USC IRB) and written informed consent was obtained for each participant (Fig 3).

An early-pregnancy visit was conducted in the first or early second trimester, during which participants completed administered questionnaires, and were measured for height and weight. Height was measured using a Perspectives Enterprises Model PE-AIM-101 stadiometer and two measurements are averaged for each participant. Weight was measured using a Tanita digital scale accurate to 5g and two measurements are averaged for each participant. For both height and weight, a third measurement was taken if the first two measurements differed by more than 1cm or 0.05kg, according to PhenX protocol [26].

At this time, a plasma sample was also collected from each consenting participant. A visit in the third trimester was conducted in a similar manner. miRNA analyses were competed after delivery to ensure that miRNA measures from paired early- and late- pregnancy samples from the same participant were measured in the same batch.

GA at birth was determined using a hierarchy of the available data: (1) ultrasound measurement of crown-rump length in first trimester (<14 weeks GA, n = 208); (2) trimester ultrasound measurement of fetal biparietal diameter in the second trimester (<28 weeks GA, n = 96). (3) obstetric clinical estimate abstracted from the participant’s medical records (n = 45). (4) date of the participant’s self-reported last menstrual period (n = 2). Participants with no available GA at birth information were excluded from this study (n = 6). GA at biospecimen collection was back-calculated using estimated GA at birth and the difference between date of collection and date of birth.

### miRNA extraction

Blood samples were collected in EDTA vacutainers, transported on ice, and were processed within 2 hours. Plasma aliquots (500μl) were stored at -80°C. Aliquots for miRNA extraction were selected from early (mean = 13.6 weeks, sd = 4.2) and late (mean = 31.7 weeks, sd = 2.0) pregnancy, as available. EV-associated miRNA was extracted from each plasma aliquot using the Qiagen ExoRNeasy kit following the manufacturer’s instructions, with a second addition of 90μL chloroform and an additional round of phase separation. The purified miRNA was frozen at -80°C until quantification.

### Quantification

Samples were initially quantified by BioAnalyzer with the small RNA kit (Agilent Technologies, Inc. USA). If the sample contained a sufficient amount of miRNA (>100 pg/μl), 3 μl of the purified miRNA was prepared using the NanoString NCounter with Human v3 miRNA expression assay (Nanostring Technologies, Inc.) with a modified protocol. Rather than the 10:1 dilution called for after addition of the hybridized probes, samples were diluted 1:2 and quantified following the rest of the recommended NanoString protocol. Replicate samples (n = 6) were run to address possible technical variation across chips. However, no significant differences were observed in miRNA counts across replicates (Wilcoxon Rank Sum, P = 0.49, S1 Fig in S1 File).

### Statistical analyses

RCC files measuring 800 miRNA for each sample were converted to text files using NSolver (v. 4.0.70, Nanostring Technologies) for downstream processing. miRNA counts were log_2_ transformed, and counts from both early and late pregnancy were normalized together in R (v. 4.0.0) using the “NanoString Norm” package (v. 1.2.1). Machine-read values were used for counts that fell below sample-specific limits of detection. Participants with high and low total miRNA counts (higher and lower than the mean total log-transformed count +/- 3 standard deviations) were dropped (8 participants), as well as those without estimated GA at birth (6 participants). For the duplicated samples used in quality control, the samples with smaller total miRNA counts were dropped (6 samples). If normalization factors flagged by NanoStringNorm were >6, individual samples were dropped (1 sample). A total of 491 total samples representing 337 unique participants were retained. Sample-specific background correction was conducted, calculated using the mean +1.5SD of negative controls. One hundred thirty miRNA had values above this sample-specific background threshold in at least 50% of samples (in either the first or third trimester) and were selected for further analysis. A Principal Components Analysis was conducted using the stats package in R [72] to identify a minimal set of covariates correlated with the first 4 miRNA PCs and to prioritize inclusion in hierarchical linear regression models, based on correlations (FDR P<0.05) (S2 Fig in S1 File).

Hierarchical linear regression models with random intercepts for subject ID and Nanostring chip were used to investigate the associations between log_2_-counts for each of the 132 miRNA and GA. Multiple testing correction was completed with False Discovery Rate (FDR) threshold of 0.05. Valid samples were required to have an associated GA at time of collection, following the selection criteria listed above. Other covariates were selected based on either *a priori* criteria, including socioeconomic status (SES) variables, or significant correlation with the first 4 PCs. Final covariates included: maternal age at 12 weeks of pregnancy, recruitment site, study entry time (before or after 20 weeks gestation), ethnicity/race (US-born White Hispanic, Foreign-born white Hispanic, other Hispanic, Black non-Hispanic, other non-Hispanic), birth order (1^st^ baby or 2^nd^ and higher), fetal sex, preferred language (English or Spanish), maternal education (less than high school, graduated high school, some college or completed college), pre-pregnancy BMI (continuous), with random effects for participant ID and chip, to account for participants providing two samples and possible batch effects. Maternal age at 12 weeks was determined using the best available GA at birth information, birth date, and maternal date of birth. Race and ethnicity were combined to better reflect the wide range of Hispanic participants.

All statistical analyses were conducted in R (4.0.0) [72] using the NLME package (3.1–143) [73]. Samples with missing values for covariates were excluded for all analyses. The main analysis used all available data (n = 449 observations). A sensitivity analysis was also conducted restricting to the subset of participants who contributed plasma samples at each timepoint (n = 296 observations). A second sensitivity analysis excluded preterm births (<37 weeks, n = 399 observations). Analyses stratified by fetal sex were also performed (n = 173 male, 161 female).

### Pathway analyses

miRNA found to be significantly (FDR P<0.05) associated with GA were selected for downstream pathway analysis. Some Nanostring miRNA targets included multiple miRNAs (e.g. hsa-miR-106a-5p+hsa-miR-17-5p), so all unique miRNA IDs were used for these cases. MirDIP version 4.1.11.1, Database version 4.1.0.3 [74] was used to predict gene targets and restricted to genes with a minimum average score >0.6. Pathway enrichment was examined using PANTHER [75–77] and all human genes in the database. Overrepresentation tests for Gene Ontology Biological Process (GOslim BP) and Molecular Function (GOslim MF) as well as PANTHER pathways were performed using Fisher’s exact test and p-values were FDR-adjusted. Number of genes in each GO category were exported from PANTHER and plotted in R with package ggplot2 [78].

## Supporting information

S1 File (DOCX)
