# Supplementary material for: Extracellular vesicle-enriched miRNA profiles across pregnancy in the MADRES cohort
Source: PLoS One. 2021 May 12;16(5):e0251259. doi: 10.1371/journal.pone.0251259 (PMC8115775; doi:10.1371/journal.pone.0251259)

**S1 File**

**Analysis of duplicate samples**. Six miRNA samples from the same collection were duplicated on Nanostring chips to investigate technical variation in quantification. Repeated measures for each quantified miRNA (n=130 miRNA, each across 6 samples) were found to be similar (Wilcoxon Rank Sum, P=0.49). In general, the miRNA counts for repeated samples were very similar.


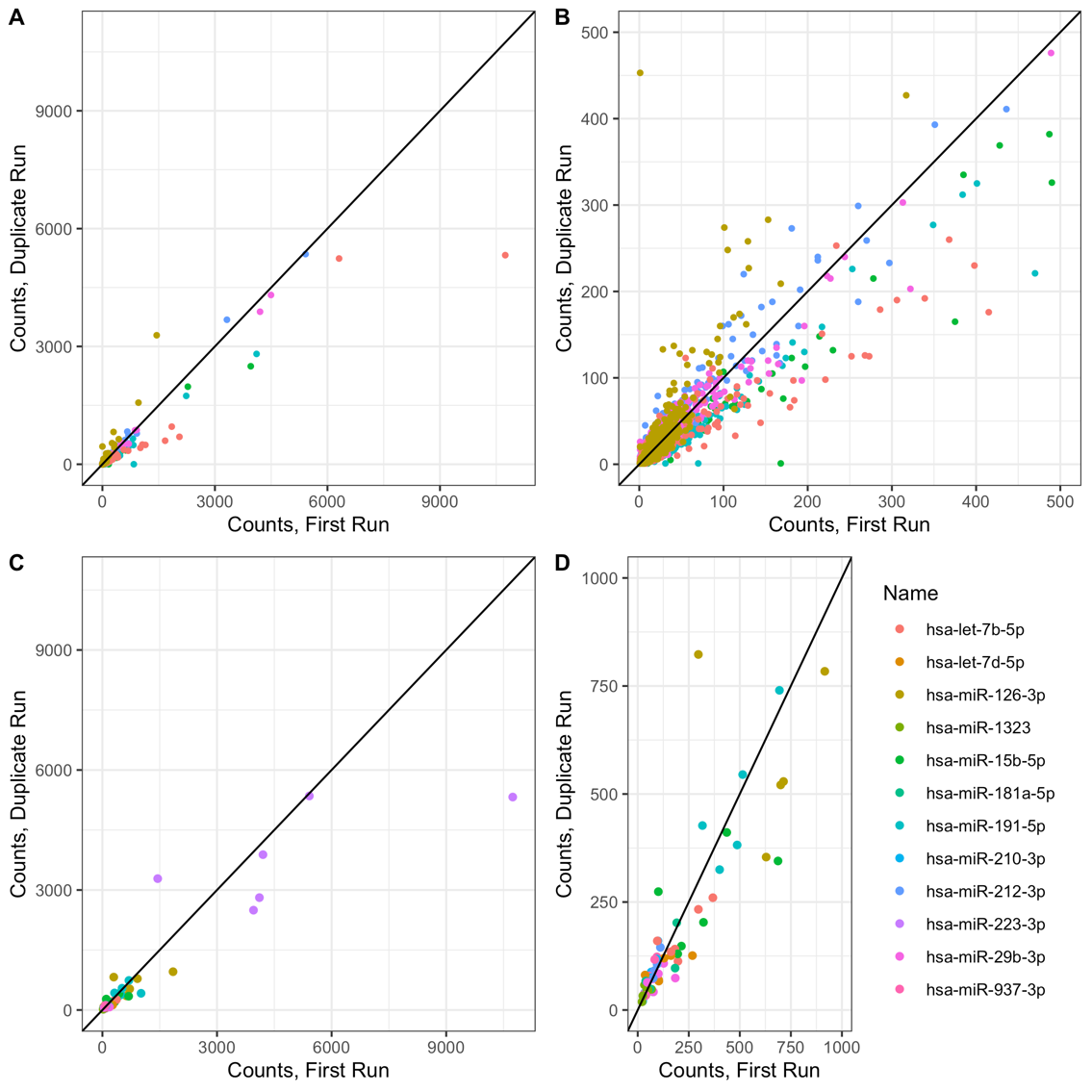
**S1 Fig.** **Plots of repeated miRNA measures.** Samples from each person are indicated by color, with most falling near the y=x line. A. All miRNA counts shown, B. miRNA with counts between 0 and 500, C. Top 12 miRNA associated with GA, D. Top 12 miRNA with counts falling between 0 and 500.

**Principal Components Analysis for selection of covariates**

Normalized counts were used to analyze the correlation of the first 4 PCs, representing 90% of the variance (Fig S-2,A), with the covariates considered in the model (Fig S-2,B).


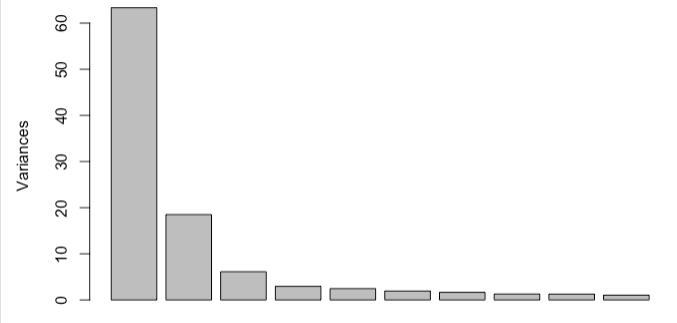

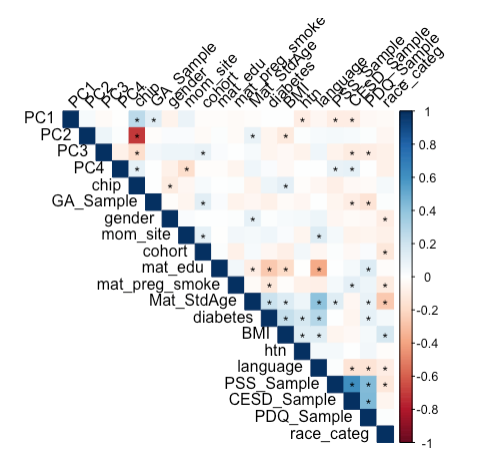
**S2 Fig. PCA Analysis.** A. Scree plot of first 10 PCs. B. Correlation plot of major covariates with the first 4 PCs. (*) indicates significant correlation (FDR P<0.05)
A.


B.

**S1 Table. GA-Associated miRNAs.** The table also lists the number of samples for which the miRNA was counted above the sample-specific limit of detection (LOD).

| **Name** | **Beta Coefficient (Counts per week GA)** | **Raw p-value** | **FDR adj. p-value** | **Number of Samples with miRNA above LOD (out of 492)** |
| --- | --- | --- | --- | --- |
| hsa-miR-126-3p | -1.28 | 1.27E-09 | 1.65E-07 | 418 |
| hsa-miR-181a-5p | -1.18 | 7.0066E-08 | 4.55E-06 | 479 |
| hsa-miR-15b-5p | -1.29 | 3.4177E-07 | 1.48E-05 | 311 |
| hsa-miR-937-3p | 1.26 | 1.7728E-06 | 5.76E-05 | 358 |
| hsa-miR-1323 | 1.1 | 3.2847E-06 | 6.93E-05 | 490 |
| hsa-let-7b-5p | -0.56 | 4.2637E-06 | 6.93E-05 | 454 |
| hsa-let-7d-5p | -0.94 | 3.9479E-06 | 6.93E-05 | 491 |
| hsa-miR-223-3p | -1.25 | 2.8148E-06 | 6.93E-05 | 430 |
| hsa-miR-29b-3p | -0.83 | 6.7004E-06 | 9.68E-05 | 490 |
| hsa-miR-191-5p | -1.17 | 7.5954E-06 | 9.87E-05 | 284 |
| hsa-miR-210-3p | 1.37 | 1.0771E-05 | 1.24E-04 | 359 |
| hsa-miR-212-3p | 1.04 | 1.2391E-05 | 0.00012 | 452 |
| hsa-let-7i-5p | -0.94 | 1.2053E-05 | 0.00012 | 328 |
| hsa-miR-15a-5p | -1.09 | 1.9532E-05 | 0.00012 | 350 |
| hsa-miR-188-5p | 1.23 | 2.4488E-05 | 0.00018 | 491 |
| hsa-miR-142-3p | -0.99 | 2.6036E-05 | 0.00020 | 491 |
| hsa-miR-199a-3p + hsa-miR-199b-3p | -1.15 | 2.6513E-05 | 0.00020 | 257 |
| hsa-miR-4707-5p | 0.96 | 3.3353E-05 | 0.00020 | 285 |
| hsa-let-7a-5p | -4.16 | 3.3193E-05 | 0.00023 | 300 |
| hsa-miR-106a-5p + hsa-miR-17-5p | -0.81 | 3.5353E-05 | 0.00023 | 304 |
| hsa-miR-1272 | 1.57 | 3.8045E-05 | 0.00023 | 328 |
| hsa-miR-525-5p | 0.92 | 4.3013E-05 | 0.00024 | 282 |
| hsa-miR-199b-5p | 1.3 | 5.9511E-05 | 0.00025 | 254 |
| hsa-miR-197-5p | 0.89 | 9.6501E-05 | 0.00034 | 471 |
| hsa-miR-1290 | 0.8 | 0.00011 | 0.00052 | 478 |
| hsa-miR-374a-5p | -0.74 | 0.00011 | 0.00055 | 332 |
| hsa-miR-302b-3p | 0.92 | 0.00012 | 0.00055 | 365 |
| hsa-miR-574-5p | 0.76 | 0.00015 | 0.00058 | 428 |
| hsa-miR-1255a | 0.86 | 0.00021 | 0.00069 | 272 |
| hsa-miR-34a-5p | 0.85 | 0.00021 | 0.00090 | 436 |
| hsa-miR-548e-5p | 0.8 | 0.00028 | 0.00090 | 257 |
| hsa-miR-526a +  hsa-miR-518c-5p +  hsa-miR-518d-5p | 0.85 | 0.00029 | 0.00117 | 304 |
| hsa-miR-608 | 0.89 | 0.00031 | 0.00119 | 424 |
| hsa-miR-183-5p | 0.8 | 0.00036 | 0.00121 | 372 |
| hsa-miR-584-3p | 0.8 | 0.00043 | 0.00139 | 481 |
| hsa-miR-320e | 1.13 | 0.0005 | 0.00158 | 257 |
| hsa-miR-138-5p | 1.19 | 0.00053 | 0.00180 | 423 |
| hsa-miR-1285-5p | 0.7 | 0.00052 | 0.00180 | 321 |
| hsa-miR-331-3p | 0.89 | 0.00065 | 0.00180 | 326 |
| hsa-miR-1972 | 0.75 | 0.00079 | 0.00215 | 277 |
| hsa-miR-873-3p | 0.83 | 0.00106 | 0.00256 | 377 |
| hsa-miR-1295a | 0.74 | 0.00101 | 0.00313 | 374 |
| hsa-miR-1305 | 0.71 | 0.00105 | 0.00313 | 423 |
| hsa-miR-186-5p | 0.55 | 0.00105 | 0.00313 | 258 |
| hsa-miR-514a-3p | 0.74 | 0.00116 | 0.00313 | 459 |
| hsa-miR-146a-5p | -0.69 | 0.00136 | 0.00335 | 311 |
| hsa-miR-584-5p | 0.74 | 0.00152 | 0.00385 | 390 |
| hsa-miR-301a-5p | 0.73 | 0.00162 | 0.00421 | 432 |
| hsa-miR-548y | 0.71 | 0.0016 | 0.00429 | 343 |
| hsa-miR-887-5p | 0.74 | 0.00165 | 0.00429 | 458 |
| hsa-miR-93-5p | -0.64 | 0.00186 | 0.00430 | 287 |
| hsa-miR-1236-3p | 0.71 | 0.00212 | 0.00473 | 263 |
| hsa-miR-328-5p | 0.71 | 0.00226 | 0.00530 | 491 |
| hsa-miR-23a-3p | -0.69 | 0.00234 | 0.00555 | 266 |
| hsa-miR-1973 | 0.75 | 0.00246 | 0.00563 | 462 |
| hsa-miR-548ah-5p | 0.7 | 0.00309 | 0.00583 | 278 |
| hsa-miR-20a-5p +  hsa-miR-20b-5p | -0.58 | 0.00341 | 0.00718 | 304 |
| hsa-miR-585-3p | 0.72 | 0.00403 | 0.00778 | 263 |
| hsa-miR-181a-3p | 0.71 | 0.00456 | 0.00904 | 383 |
| hsa-miR-10b-5p | 0.62 | 0.00474 | 0.01005 | 249 |
| hsa-miR-96-5p | 0.64 | 0.00498 | 0.01028 | 314 |
| hsa-miR-939-5p | 0.64 | 0.0054 | 0.01062 | 257 |
| hsa-miR-208a-3p | 0.67 | 0.00569 | 0.01133 | 382 |
| hsa-miR-1296-3p | 0.64 | 0.00578 | 0.01174 | 437 |
| hsa-miR-199a-5p | -0.47 | 0.00648 | 0.01174 | 247 |
| hsa-miR-764 | 0.66 | 0.00683 | 0.01295 | 338 |
| hsa-miR-1262 | 0.65 | 0.00714 | 0.01345 | 261 |
| hsa-miR-495-5p | 0.68 | 0.00744 | 0.01385 | 315 |
| hsa-miR-548ar-3p | 0.6 | 0.00778 | 0.01423 | 446 |
| hsa-miR-543 | 0.42 | 0.0077 | 0.01445 | 255 |
| hsa-miR-98-5p | -0.49 | 0.00928 | 0.01445 | 266 |
| hsa-miR-18a-5p | 0.4 | 0.01089 | 0.01698 | 453 |
| hsa-miR-130a-3p | -0.66 | 0.01265 | 0.01966 | 315 |
| hsa-miR-299-5p | 0.54 | 0.01342 | 0.02252 | 350 |
| hsa-miR-607 | 0.57 | 0.01372 | 0.02358 | 438 |
| hsa-miR-30e-5p | 0.47 | 0.01544 | 0.02378 | 340 |
| hsa-miR-1258 | 0.58 | 0.0157 | 0.02640 | 226 |
| hsa-miR-151a-3p | -0.46 | 0.0168 | 0.02650 | 317 |
| hsa-miR-1268b | 0.55 | 0.01756 | 0.02800 | 346 |
| hsa-miR-221-3p | -0.47 | 0.01776 | 0.02851 | 397 |
| hsa-miR-21-5p | -0.48 | 0.01769 | 0.02851 | 265 |
| hsa-miR-1307-3p | 0.53 | 0.0185 | 0.02851 | 270 |
| hsa-miR-369-3p | 0.58 | 0.01933 | 0.02933 | 411 |
| hsa-miR-3144-3p | 0.92 | 0.01964 | 0.03027 | 290 |
| hsa-miR-19b-3p | -0.48 | 0.02405 | 0.03040 | 421 |
| hsa-miR-1827 | 0.53 | 0.02776 | 0.03678 | 415 |
| hsa-miR-1257 | 0.47 | 0.02744 | 0.04148 | 298 |
| hsa-miR-656-3p | 0.46 | 0.03198 | 0.04148 | 398 |
| hsa-miR-26a-5p | 0.45 | 0.03239 | 0.04724 | 397 |
| hsa-miR-1197 | 0.47 | 0.03345 | 0.04731 | 290 |

**S3 Fig. Volcano plot of miRNA associated with GA, scaled to 18 weeks of pregnancy.** Sensitivity analysis, restricting to participants with both early and late pregnancy samples. Red points indicate FDR-adjusted significant miRNA p<0.05. Red vertical dashed lines indicate -10% and 10% change over 18 weeks.


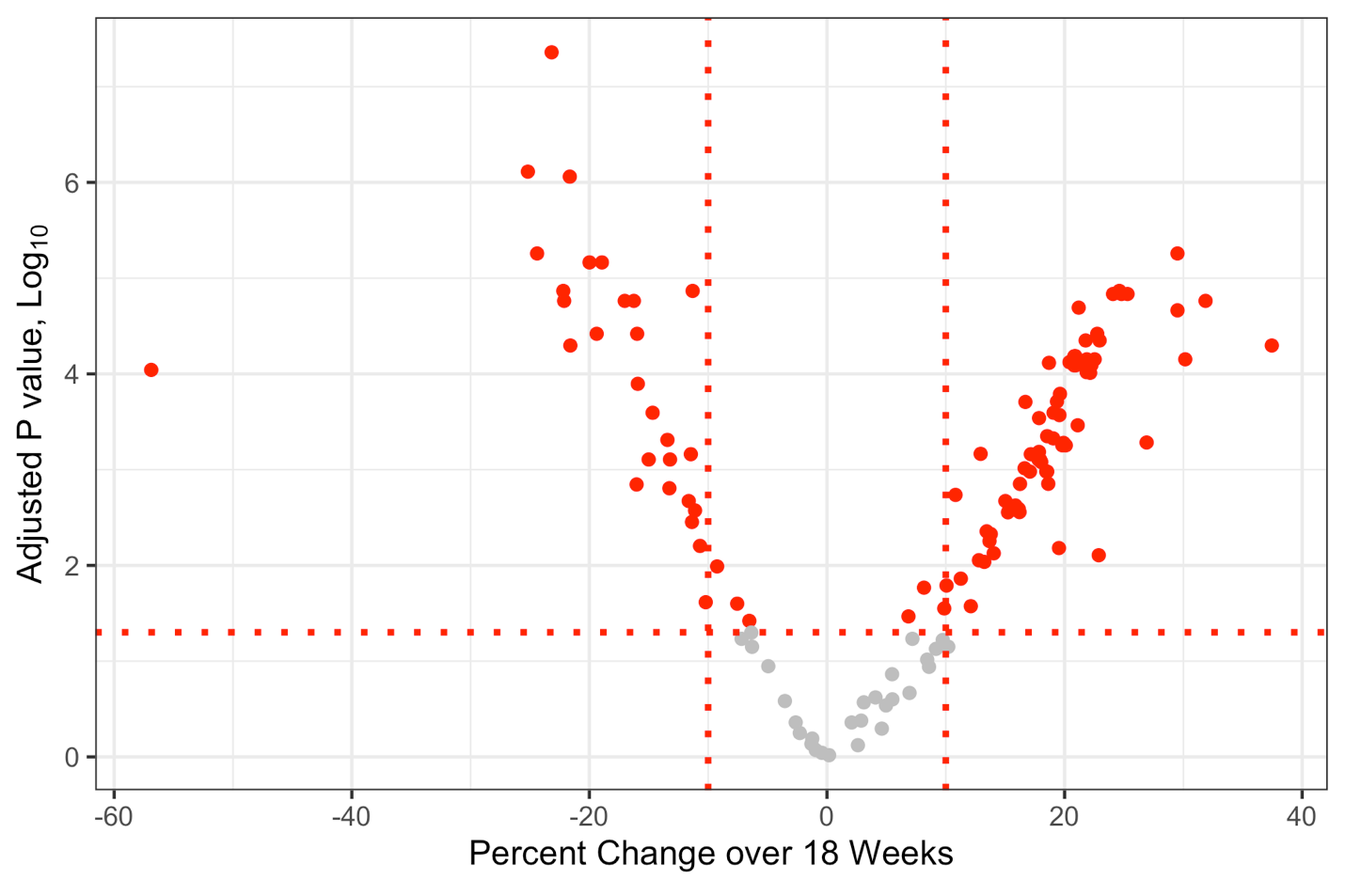


**S2 Table.** **miRNA significantly associated with GA from analyses stratified by fetal sex.**

|  | **Females** |  | **Males** | |
| --- | --- | --- | --- | --- |
| **miRNA Name** | **Beta coefficient**  **(Counts per  week GA)** | **FDR adj.  p-value** | **Beta coefficient**  **(Counts per  week GA)** | **FDR adj.  p-value** |
| hsa-miR-126-3p | -1.3 | 0.00194 | -1.26 | 0.00194 |
| hsa-let-7b-5p | -0.683 | 0.00408 | -0.464 | 0.0314 |
| hsa-miR-223-3p | -1.34 | 0.00952 | -1.17 | 0.0126 |
| hsa-miR-181a-5p | -1.01 | 0.0126 | -1.31 | 0.00194 |
| hsa-miR-191-5p | -1.24 | 0.0126 | -1.14 | 0.0146 |
| hsa-miR-29b-3p | -0.895 | 0.0126 | -0.743 | 0.0183 |
| hsa-miR-526a +  hsa-miR-518c-5p +  hsa-miR-518d-5p | 0.991 | 0.0126 | NA | NA |
| hsa-miR-15b-5p | -1.15 | 0.0137 | -1.46 | 0.0022 |
| hsa-miR-199a-3p + hsa-miR-199b-3p | -1.23 | 0.0153 | -1.11 | 0.0208 |
| hsa-miR-197-5p | 0.977 | 0.0153 | 0.869 | 0.0352 |
| hsa-miR-212-3p | 1.09 | 0.0154 | 0.959 | 0.0183 |
| hsa-miR-574-5p | 0.858 | 0.0157 | 0.691 | 0.046 |
| hsa-let-7d-5p | -0.904 | 0.0183 | -0.973 | 0.00775 |
| hsa-miR-873-3p | 0.95 | 0.0187 | NA | NA |
| hsa-miR-142-3p | -1.05 | 0.0193 | -0.956 | 0.0177 |
| hsa-let-7a-5p | -4.32 | 0.02 | -4.06 | 0.0183 |
| hsa-miR-937-3p | 1.1 | 0.0224 | 1.39 | 0.00415 |
| hsa-miR-183-5p | 0.914 | 0.0228 | 0.747 | 0.0464 |
| hsa-miR-374a-5p | -0.774 | 0.0243 | -0.725 | 0.0299 |
| hsa-miR-1323 | 0.937 | 0.0259 | 1.3 | 0.00415 |
| hsa-miR-320e | 1.23 | 0.0259 | NA | NA |
| hsa-miR-199b-5p | 1.23 | 0.0309 | 1.33 | 0.0208 |
| hsa-miR-608 | 0.95 | 0.0311 | 0.822 | 0.0487 |
| hsa-miR-210-3p | 1.14 | 0.0314 | 1.56 | 0.00911 |
| hsa-miR-1272 | 1.33 | 0.0314 | 1.75 | 0.0153 |
| hsa-miR-302b-3p | 0.847 | 0.0314 | 1 | 0.0259 |
| hsa-miR-34a-5p | 0.854 | 0.0314 | 0.852 | 0.0366 |
| hsa-miR-93-5p | -0.776 | 0.0335 | NA | NA |
| hsa-miR-1285-5p | 0.752 | 0.0352 | 0.696 | 0.046 |
| hsa-miR-585-3p | 0.934 | 0.0366 | NA | NA |
| hsa-miR-1258 | 0.861 | 0.0403 | NA | NA |
| hsa-miR-138-5p | 1.24 | 0.0416 | NA | NA |
| hsa-miR-4707-5p | 0.827 | 0.0419 | 1.07 | 0.0126 |
| hsa-miR-1236-3p | 0.867 | 0.0431 | NA | NA |
| hsa-miR-1305 | 0.821 | 0.0431 | NA | NA |
| hsa-miR-331-3p | 0.956 | 0.0431 | NA | NA |
| hsa-miR-188-5p | 0.965 | 0.0435 | 1.45 | 0.0102 |
| hsa-miR-96-5p | 0.787 | 0.044 | NA | NA |
| hsa-miR-887-5p | 0.84 | 0.0441 | NA | NA |
| hsa-miR-584-3p | 0.785 | 0.0443 | 0.81 | 0.0425 |
| hsa-miR-1295a | 0.745 | 0.0443 | NA | NA |
| hsa-miR-328-5p | 0.855 | 0.0443 | NA | NA |
| hsa-miR-15a-5p | -0.901 | 0.047 | -1.27 | 0.00692 |
| hsa-miR-1290 | 0.723 | 0.047 | 0.893 | 0.0146 |
| hsa-miR-106a-5p+ hsa-miR-17-5p | -0.685 | 0.0487 | -0.95 | 0.00739 |
| hsa-miR-525-5p | 0.779 | 0.0487 | 1.11 | 0.00739 |
| hsa-miR-186-5p | 0.537 | 0.0487 | 0.608 | 0.0443 |
| hsa-miR-23a-3p | -0.773 | 0.0487 | NA | NA |
| hsa-let-7i-5p | NA | NA | -1.16 | 0.00439 |
| hsa-miR-1255a | NA | NA | 1.07 | 0.0126 |
| hsa-miR-18a-5p | NA | NA | 0.674 | 0.0126 |
| hsa-miR-1972 | NA | NA | 1.03 | 0.0126 |
| hsa-miR-20a-5p+ hsa-miR-20b-5p | NA | NA | -0.938 | 0.0126 |
| hsa-miR-548e-5p | NA | NA | 0.938 | 0.0183 |
| hsa-miR-939-5p | NA | NA | 0.894 | 0.0208 |
| hsa-miR-146a-5p | NA | NA | -0.854 | 0.0284 |
| hsa-miR-514a-3p | NA | NA | 0.848 | 0.0352 |
| hsa-miR-10b-5p | NA | NA | 0.812 | 0.0382 |
| hsa-miR-764 | NA | NA | 0.899 | 0.0382 |
| hsa-miR-543 | NA | NA | 0.572 | 0.0435 |
| hsa-miR-584-5p | NA | NA | 0.883 | 0.0443 |
| hsa-miR-607 | NA | NA | 0.748 | 0.0443 |
| hsa-miR-301a-5p | NA | NA | 0.763 | 0.0464 |

Note: NA indicates that the miRNA was not significantly associated with gestational age.

**S4 Figure. Venn Diagram of sex-stratified miRNA.** 33 miRNA were shared between analyses stratified by sex for association with GA. Fifteen miRNA were significantly associated with GA for females only, and 15 different miRNA for males only. All of these were also significant in the main analysis with data from both sexes.

**
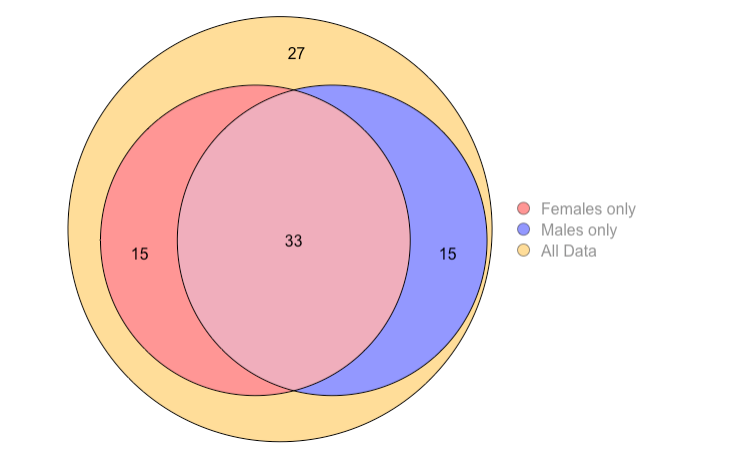
**

**S3 Table.** Table comparing the participant demographics for those contributing 1 or 2 samples.

|  |  | Participants with  2 Samples | | Participants with  1 Sample | |
| --- | --- | --- | --- | --- | --- |
|  |  | n | Mean (SD) | n | Mean (SD) |
| Maternal Age | Standardized at 12 weeks | 154 | 28.5 yrs (5.9) | 184 | 28.6 yrs (6.2) |
|  |  |  |  |  |  |
|  | Early pregnancy samples | 154 | 13.6 wks (4.2) | 39 | 13.3 wks (4.6) |
|  | Late pregnancy samples | 154 | 31.9 wks (1.8) | 145 | 31.4 wks (2.1) |
|  |  |  |  |  |  |
|  |  | n | Percent | n | Percent |
| Fetal Sex | Female | 65 | 43.3% | 93 | 51.4% |
|  | Male | 85 | 56.7% | 88 | 48.6% |
|  |  |  |  |  |  |
| Parity | Nulliparous | 50 | 32.5% | 66 | 38.6% |
|  | Primiparous or higher | 104 | 67.5% | 105 | 61.4% |
|  |  |  |  |  |  |
| Language | English | 90 | 60.4% | 101 | 64.3% |
|  | Spanish | 59 | 39.6% | 56 | 35.7% |
|  |  |  |  |  |  |
| Race/Ethnicity  *(p<0.005) | US-Born White Hispanic | 51 | 33.1% | 63 | 34.4% |
|  | Foreign-Born White Hispanic | 76 | 49.4% | 63 | 34.4% |
|  | Other Hispanic | 4 | 2.6% | 20 | 10.9% |
|  | Black Non-Hispanic | 15 | 9.7% | 19 | 10.4% |
|  | Non-Hispanic | 8 | 5.2% | 18 | 9.8% |
|  |  |  |  |  |  |
| Education | Less than 12th grade (Did not finish high school) | 39 | 25.3% | 60 | 32.8% |
|  | Completed 12th grade (Graduated high school) | 53 | 34.4% | 44 | 24.0% |
|  | Some college or completed college | 62 | 40.2% | 79 | 43.2% |
|  |  |  |  |  |  |
| Pre-pregnancy  BMI | Underweight or Normal (< 25 kg/m^2^) | 49 | 31.8% | 58 | 37.7% |
|  | Overweight  (25 kg/m^2^ – 29.9 kg/m^2^) | 50 | 32.5% | 61 | 39.6% |
|  | Class 1-3 obese (> 30 kg/m^2^) | 55 | 35.7% | 64 | 41.6% |

Note: Statistical tests for differences between these groups (T-test: maternal age; Chi-square test: fetal sex, birth order, language, education and pre-pregnancy BMI; Fisher’s exact test: race) were not significant for maternal age, fetal sex, birth order, language, education and BMI. There was a significant difference in race between the groups (p<0.005).

**S4 Table. miRNA significantly associated with GA from the main analysis and term birth (>37 weeks) sensitivity study.** Eighty six miRNA were significant in both studies, while only 4 miRNA lost significance in the sensitivity study relative to the main study. Eight miRNA were significantly associated with GA in the sensitivity study but not in the main study.

|  | **Preterm birth excluded** | | **All data** | |
| --- | --- | --- | --- | --- |
| **miRNA Name** | **Beta coefficient**  **(Counts per  week GA)** | **FDR adj.  p-value** | **Beta coefficient**  **(Counts per  week GA)** | **FDR adj.  p-value** |
| hsa-let-7i-5p | -0.961 | 0.000154 | -0.942 | 0.000124 |
| hsa-miR-210-3p | 1.4 | 0.000154 | 1.37 | 0.000124 |
| hsa-miR-212-3p | 1.05 | 0.000166 | 1.04 | 0.000124 |
| hsa-miR-15a-5p | -1.07 | 0.000596 | -1.09 | 0.000181 |
| hsa-miR-188-5p | 1.2 | 0.000193 | 1.23 | 0.000203 |
| hsa-miR-199a-3p + hsa-miR-199b-3p | -1.18 | 0.000398 | -1.15 | 0.000203 |
| hsa-miR-142-3p | -0.996 | 5.00E-04 | -0.991 | 0.000203 |
| hsa-let-7a-5p | -4.61 | 0.000154 | -4.16 | 0.000228 |
| hsa-miR-4707-5p | 1.03 | 0.000193 | 0.959 | 0.000228 |
| hsa-miR-106a-5p+hsa-miR-17-5p | -0.864 | 0.000277 | -0.811 | 0.00023 |
| hsa-miR-1272 | 1.55 | 0.000263 | 1.57 | 0.000236 |
| hsa-miR-525-5p | 0.912 | 0.000565 | 0.919 | 0.000254 |
| hsa-miR-199b-5p | 1.35 | 0.000322 | 1.3 | 0.000336 |
| hsa-miR-197-5p | 0.915 | 0.000655 | 0.889 | 0.000523 |
| hsa-miR-1290 | 0.873 | 0.000565 | 0.805 | 0.000549 |
| hsa-miR-374a-5p | -0.78 | 0.000596 | -0.737 | 0.000549 |
| hsa-miR-302b-3p | 0.861 | 0.00142 | 0.925 | 0.000579 |
| hsa-miR-574-5p | 0.835 | 5.00E-04 | 0.759 | 0.000688 |
| hsa-miR-1255a | 0.908 | 0.000655 | 0.861 | 0.000901 |
| hsa-miR-34a-5p | 0.822 | 0.00269 | 0.853 | 0.000901 |
| hsa-miR-548e-5p | 0.87 | 0.000801 | 0.799 | 0.00117 |
| hsa-miR-526a + hsa-miR-518c-5p + hsa-miR-518d-5p | 0.824 | 0.00313 | 0.845 | 0.00119 |
| hsa-miR-608 | 0.917 | 0.000788 | 0.888 | 0.00121 |
| hsa-miR-183-5p | 0.87 | 0.00104 | 0.795 | 0.00139 |
| hsa-miR-584-3p | 0.882 | 0.00104 | 0.8 | 0.00158 |
| hsa-miR-1285-5p | 0.811 | 0.000596 | 0.703 | 0.0018 |
| hsa-miR-138-5p | 1.29 | 0.000738 | 1.19 | 0.0018 |
| hsa-miR-320e | 1.1 | 0.00487 | 1.13 | 0.0018 |
| hsa-miR-331-3p | 0.973 | 0.00142 | 0.893 | 0.00215 |
| hsa-miR-1972 | 0.774 | 0.00269 | 0.753 | 0.00256 |
| hsa-miR-1295a | 0.81 | 0.00254 | 0.735 | 0.00313 |
| hsa-miR-186-5p | 0.618 | 0.00254 | 0.554 | 0.00313 |
| hsa-miR-1305 | 0.768 | 0.00282 | 0.709 | 0.00313 |
| hsa-miR-873-3p | 0.769 | 0.00745 | 0.832 | 0.00313 |
| hsa-miR-514a-3p | 0.698 | 0.00717 | 0.736 | 0.00335 |
| hsa-miR-146a-5p | -0.701 | 0.00399 | -0.691 | 0.00385 |
| hsa-miR-584-5p | 0.795 | 0.00225 | 0.742 | 0.00421 |
| hsa-miR-548y | 0.743 | 0.00427 | 0.708 | 0.00429 |
| hsa-miR-301a-5p | 0.758 | 0.00554 | 0.728 | 0.00429 |
| hsa-miR-887-5p | 0.784 | 0.00497 | 0.744 | 0.0043 |
| hsa-miR-93-5p | -0.667 | 0.00513 | -0.641 | 0.00473 |
| hsa-miR-1236-3p | 0.73 | 0.00684 | 0.712 | 0.0053 |
| hsa-miR-328-5p | 0.74 | 0.00557 | 0.711 | 0.00555 |
| hsa-miR-23a-3p | -0.718 | 0.00711 | -0.694 | 0.00563 |
| hsa-miR-1973 | 0.77 | 0.00622 | 0.747 | 0.00583 |
| hsa-miR-548ah-5p | 0.792 | 0.00498 | 0.704 | 0.00718 |
| hsa-miR-20a-5p + hsa-miR-20b-5p | -0.57 | 0.00554 | -0.576 | 0.00778 |
| hsa-miR-585-3p | 0.836 | 0.00501 | 0.724 | 0.00904 |
| hsa-miR-181a-3p | 0.739 | 0.00765 | 0.708 | 0.0101 |
| hsa-miR-10b-5p | 0.717 | 0.00554 | 0.62 | 0.0103 |
| hsa-miR-96-5p | 0.649 | 0.0133 | 0.643 | 0.0106 |
| hsa-miR-939-5p | 0.718 | 0.00649 | 0.641 | 0.0113 |
| hsa-miR-208a-3p | 0.751 | 0.00832 | 0.673 | 0.0117 |
| hsa-miR-1296-3p | 0.615 | 0.0175 | 0.637 | 0.0117 |
| hsa-miR-199a-5p | -0.518 | 0.00693 | -0.467 | 0.013 |
| hsa-miR-764 | 0.722 | 0.0111 | 0.665 | 0.0134 |
| hsa-miR-1262 | 0.667 | 0.0133 | 0.647 | 0.0139 |
| hsa-miR-495-5p | 0.816 | 0.00622 | 0.685 | 0.0142 |
| hsa-miR-548ar-3p | 0.716 | 0.00622 | 0.602 | 0.0144 |
| hsa-miR-543 | 0.494 | 0.00839 | 0.424 | 0.0144 |
| hsa-miR-98-5p | -0.513 | 0.0197 | -0.49 | 0.017 |
| hsa-miR-18a-5p | 0.492 | 0.00622 | 0.396 | 0.0197 |
| hsa-miR-130a-3p | -0.672 | 0.0294 | -0.664 | 0.0225 |
| hsa-miR-299-5p | 0.635 | 0.0124 | 0.543 | 0.0236 |
| hsa-miR-607 | 0.727 | 0.00622 | 0.572 | 0.0238 |
| hsa-miR-30e-5p | 0.471 | 0.0343 | 0.47 | 0.0264 |
| hsa-miR-1258 | 0.677 | 0.0134 | 0.575 | 0.0265 |
| hsa-miR-151a-3p | -0.41 | NA | -0.463 | 0.028 |
| hsa-miR-21-5p | -0.503 | 0.0195 | -0.478 | 0.0285 |
| hsa-miR-1268b | 0.587 | 0.026 | 0.549 | 0.0285 |
| hsa-miR-221-3p | -0.495 | 0.0288 | -0.471 | 0.0285 |
| hsa-miR-1307-3p | 0.596 | 0.0214 | 0.528 | 0.0293 |
| hsa-miR-369-3p | 0.531 | NA | 0.576 | 0.0303 |
| hsa-miR-3144-3p | 1.04 | 0.0163 | 0.921 | 0.0304 |
| hsa-miR-19b-3p | -0.482 | 0.0472 | -0.484 | 0.0368 |
| hsa-miR-1257 | 0.556 | 0.0217 | 0.472 | 0.0415 |
| hsa-miR-1827 | 0.579 | 0.0385 | 0.531 | 0.0415 |
| hsa-miR-656-3p | 0.529 | 0.03 | 0.459 | 0.0472 |
| hsa-miR-26a-5p | 0.384 | NA | 0.453 | 0.0473 |
| hsa-miR-1197 | 0.489 | NA | 0.474 | 0.0483 |
| hsa-miR-15b-5p | -1.26 | 7.68E-05 | -1.29 | 1.48E-05 |
| hsa-miR-126-3p | -1.36 | 1.51E-07 | -1.28 | 1.65E-07 |
| hsa-miR-181a-5p | -1.25 | 5.95E-06 | -1.18 | 4.55E-06 |
| hsa-miR-937-3p | 1.14 | 0.000254 | 1.26 | 5.76E-05 |
| hsa-let-7b-5p | -0.589 | 0.000132 | -0.562 | 6.93E-05 |
| hsa-let-7d-5p | -0.932 | 0.000193 | -0.941 | 6.93E-05 |
| hsa-miR-1323 | 1.2 | 7.68E-05 | 1.1 | 6.93E-05 |
| hsa-miR-223-3p | -1.34 | 7.68E-05 | -1.25 | 6.93E-05 |
| hsa-miR-29b-3p | -0.886 | 0.000154 | -0.831 | 9.68E-05 |
| hsa-miR-191-5p | -1.14 | 0.000322 | -1.17 | 9.87E-05 |
| hsa-miR-376a-3p | 0.431 | 0.0141 | 0.308 | NA |
| hsa-miR-144-3p | 0.638 | 0.0245 | 0.497 | NA |
| hsa-miR-423-5p | -0.458 | 0.0309 | -0.365 | NA |
| hsa-miR-593-3p | 0.545 | 0.0311 | 0.467 | NA |
| hsa-miR-411-5p | 0.552 | 0.0398 | 0.435 | NA |
| hsa-miR-378h | 0.568 | 0.04 | 0.387 | NA |
| hsa-miR-3147 | 0.553 | 0.043 | 0.504 | NA |
| hsa-miR-612 | 0.523 | 0.0472 | 0.428 | NA |

**S5 Figure. Plots of miRNA expression by GA for participants with two samples.** The top 12 miRNA with significant associations for GA are shown. Samples from the same participant are connected by a thin line, showing that miRNA levels changed over time.


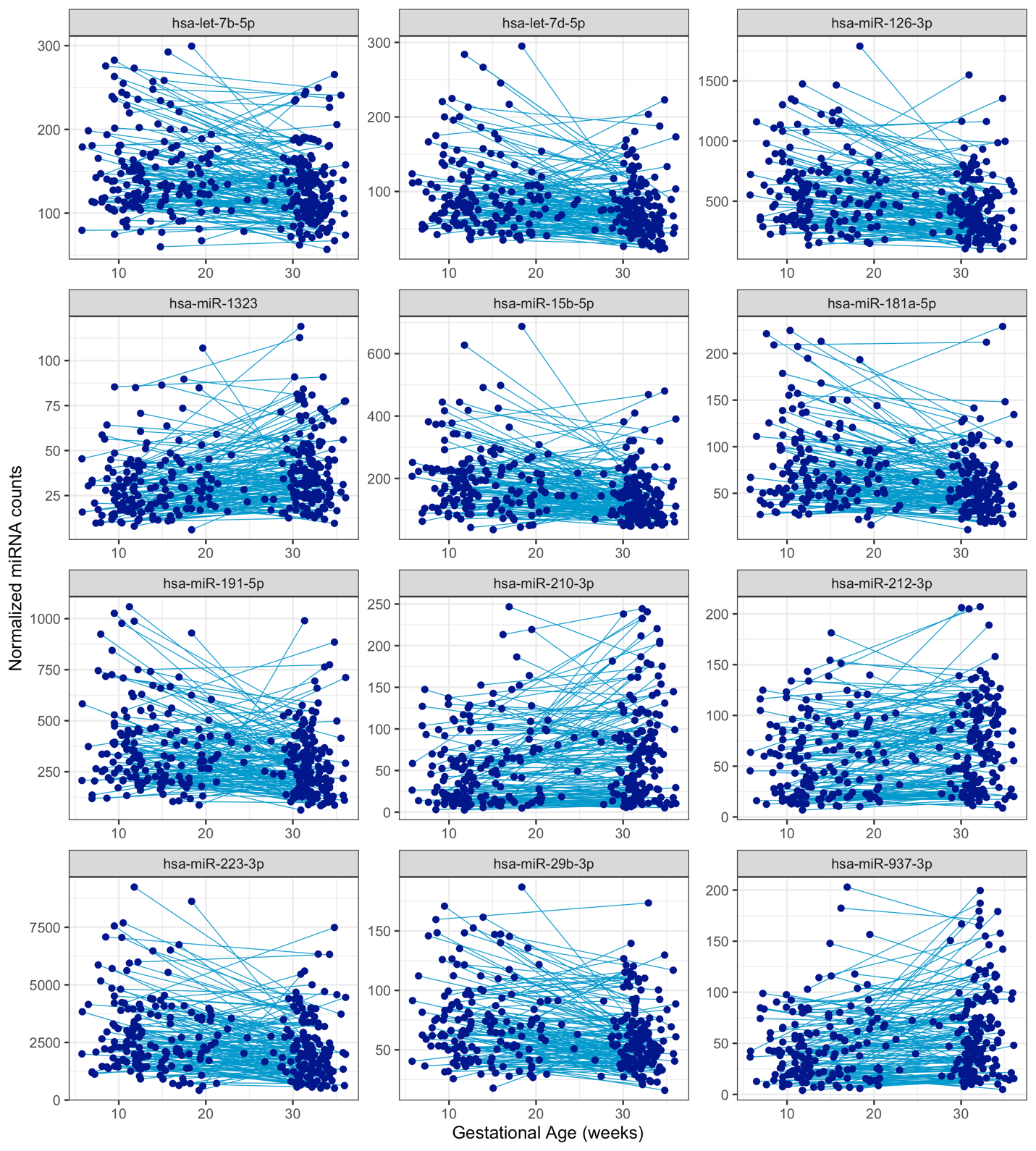

Supplement: S1 File — (DOCX) [file pone.0251259.s001.docx]
